# Analysis of genomic differences among *Clostridium botulinum *type A1 strains

**DOI:** 10.1186/1471-2164-11-725

**Published:** 2010-12-23

**Authors:** Ping-Ke Fang, Brian H Raphael, Susan E Maslanka, Shuowei Cai, Bal Ram Singh

**Affiliations:** 1 Botulinum Research Center and Department of Chemistry & Biochemistry, University of Massachusetts Dartmouth, 285 Old Westport Road, North Dartmouth, Massachusetts 02747, USA; 2 Enteric Diseases Laboratory Branch, Centers for Disease Control and Prevention, Atlanta, Georgia, USA

## Abstract

**Background:**

Type A1 *Clostridium botulinum *strains are a group of Gram-positive, spore-forming anaerobic bacteria that produce a genetically, biochemically, and biophysically indistinguishable 150 kD protein that causes botulism. The genomes of three type A1 *C. botulinum *strains have been sequenced and show a high degree of synteny. The purpose of this study was to characterize differences among these genomes and compare these differentiating features with two additional unsequenced strains used in previous studies.

**Results:**

Several strategies were deployed in this report. First, University of Massachusetts Dartmouth laboratory Hall strain (UMASS strain) neurotoxin gene was amplified by PCR and sequenced; its sequence was aligned with the published ATCC 3502 Sanger Institute Hall strain and Allergan Hall strain neurotoxin gene regions. Sequence alignment showed that there was a synonymous single nucleotide polymorphism (SNP) in the region encoding the heavy chain between Allergan strain and ATCC 3502 and UMASS strains. Second, comparative genomic hybridization (CGH) demonstrated that the UMASS strain and a strain expected to be derived from ATCC 3502 in the Centers for Disease Control and Prevention (CDC) laboratory (ATCC 3502*) differed in gene content compared to the ATCC 3502 genome sequence published by the Sanger Institute. Third, alignment of the three sequenced *C. botulinum *type A1 strain genomes revealed the presence of four comparable blocks. Strains ATCC 3502 and ATCC 19397 share the same genome organization, while the organization of the blocks in strain Hall were switched. Lastly, PCR was designed to identify UMASS and ATCC 3502* strain genome organizations. The PCR results indicated that UMASS strain belonged to Hall type and ATCC 3502* strain was identical to ATCC 3502 (Sanger Institute) type.

**Conclusions:**

Taken together, *C. botulinum *type A1 strains including Sanger Institute ATCC 3502, ATCC 3502*, ATCC 19397, Hall, Allergan, and UMASS strains demonstrate differences at the level of the neurotoxin gene sequence, in gene content, and in genome arrangement.

## Background

*Clostridium botulinum *is a Gram-positive, spore-forming anaerobic bacterium that causes the severe neuroparalytic illness in humans and animals known as botulism. There are seven serologically distinct types of botulinum neurotoxin -types A, B, C, D, E, F, and G. Comparison of 16 S rRNA sequences [1] showed that *C. botulinum *strains forms four distinct clusters that correspond to four physiological groups (I-IV), which supported the historical classification scheme based upon biochemical and biophysical parameters. Group I (proteolytic *C. botulinum*) strains produce one or sometimes two toxins of type A, B or F; Group II (non-proteolytic *C. botulinum*) strains produce toxins of type B, E, or F; Group III strains produce toxins of type C or D; and Group IV strains produce toxin of type G [2,3]. Furthermore, the toxinotypes are divided into many subtypes, which have been defined as toxin sequences differing by at least 2.6% identity at amino acid level [4]. Botulinum type A neurotoxins are divided into five subtypes termed A1, A2, A3, A4, and more recently to A5 [5]; botulinum type B neurotoxins are divided into five subtypes termed B1, B2, B3, bivalent B, and non-proteolytic botulinum B neurotoxin; botulinum type E neurotoxins are classified into six subtypes: E1, E2, E3, E4, E5, and E6 [6]; and botulinum type F neurotoxins are separated into F1 through F7 subtypes [7]. There are no known subtypes from types C, D, and G [8].

The strains of *C. botulinum *used for the production of type A therapeutic toxin (recently referred to as botulinum neuromedicine or BoNEM; Singh, 2009) are likely to originate from those isolated and preserved by Ivan C. Hall in the early 1900s. These strains, which include include several type A strains from botulism cases in the western United States [9] and both type A and B strains from isolated wounds [10], were distributed to colleges and universities throughout the world and deposited in various culture collections [11,12]. As a result, many subcultures were performed, and the strains designated as "Hall" strains may not be identical to or may differ from the original isolates as a result of long term passage.

In this communication, the genetic diversity of *C. botulinum *was further explored by comparing the genomic differences among several *C. botulinum *strains including Sanger Institute ATCC 3502 [Hall 174, GenBank: AM412317], CDC ATCC 3502 (ATCC 3502*), ATCC 19397 [GenBank: CP000726], Hall [GenBank: CP000727], Allergan, and University of Massachusetts Dartmouth laboratory Hall strain (UMASS strain), all belonging to subtype A1. The results indicated that genetic diversity existed among these subtype A1 strains including those designated simply as "Hall".

## Results

### *C. botulinum *A1 neurotoxin gene complex

The *C. botulinum *type A1 neurotoxin complex genomic cluster spans 11719 bp in ATCC 3502 [GenBank: AM412317, positions 901881 through 913599]. The identical genomic cluster was also found in four other genome sequences, whose GenBank accession numbers are CP000727 (*C. botulinum *A strain Hall), CP000726 (*C. botulinum *A strain ATCC 19397), DQ409059 (Hall A BoNT/A cluster), and AF461540 (Hall A-*hyper *BoNT/A cluster and its flanking regions).

The botulinum type A1 neurotoxin complex consists of six genes, namely, *ha70, ha17, ha33, botR, ntnh*, and *bont/A*, whose coding regions in aggregate consist of 11215 bp out of 11719 bp botulinum A neurotoxin genomic cluster (Sanger Institute ATCC 3502). Sequence alignment of this cluster with each individual gene sequence [GenBank: AF488745-AF488750] from the Allergan Hall strain [13] revealed that only two base pairs were different: one was in the region encoding the heavy chain of neurotoxin botulinum type A1, the other was in *botR *region (position 9 in AF488750, data not shown). Both are synonymous single nucleotide polymorphisms (SNP), which are not predicted to result in an amino acid change.

The botulinum neurotoxin type A1 gene (*bont/A*) from the UMASS strain was also sequenced and compared to that of ATCC 3502 and Allergan Hall strains. The UMASS sequence was identical to ATCC 3502 but different from Allergan Hall strain by one base pair (position 3591 of the neurotoxin gene, data not shown). We did not sequence the *botR *region in UMASS strain, therefore, it is unclear whether the SNP in *botR *region exists or not.

### Comparative genomic hybridization of UMASS strain

**Figure 1:**
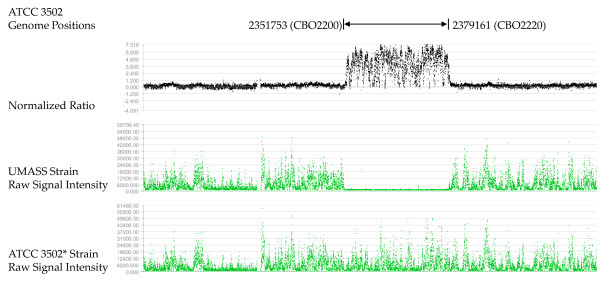
**Absence of CBO2200-CBO2220 in the UMASS strain**. The number above tracks indicates the absence region in the ATCC 3502 genome location and its corresponding Coding Sequence (CDS) location, expressed as CBO number; the middle track is the UMASS strain raw signal intensity; and the bottom track is the ATCC 3502* reference strain raw signal intensity. The top track is normalized log_2 _ratios of the fluorescence intensity of the reference strain/test strain. UMASS strain genome fragment corresponding to ATCC 3502 genome region from 2351753 (the start of CBO2200) to 2379161 (the end of CBO2220) is absent.

**Figure 2:**
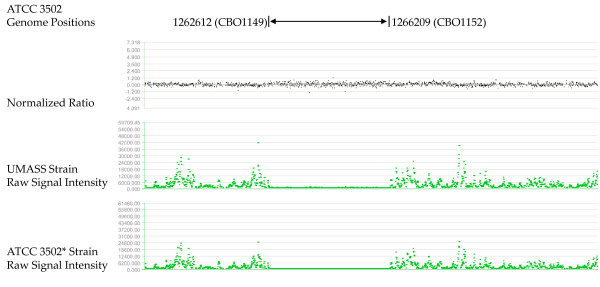
Absence of CBO1149-CBO1152 in both UMASS and ATCC 3502* strains. Tracks are laid out as those in Figure 1. Both UMASS strain and ATCC 3502* genome fragment corresponding to ATCC 3502 genome region from position 1262612 (the start of CBO1149) to position 1266209 (the end of CBO1152) are absent.

The comparative genomic hybridization (CGH) microarray featured overlapping probes covering the entire *C. botulinum *A1 strain ATCC 3502 genome sequence [GenBank: AM412317]. The ATCC 3502* strain was used as reference and the UMASS strain as test strain. The hybridization results indicated the presence of several regions that were different between UMASS strain and Sanger Institute ATCC 3502 strain (Figure 1 and Figure 2), and, in some cases, were even different between ATCC 3502* and Sanger Institute ATCC 3502 strain (Figure 2). The nature of the deleted sequence (27409 bp) in Figure 1 is unclear. The same block sequence was also found in ATCC 19397 genome but not in Hall strain genome, as retrieved through NCBI Blast server.

### Genome organizations of *C. botulinum *A1 strains

**Figure 3:**
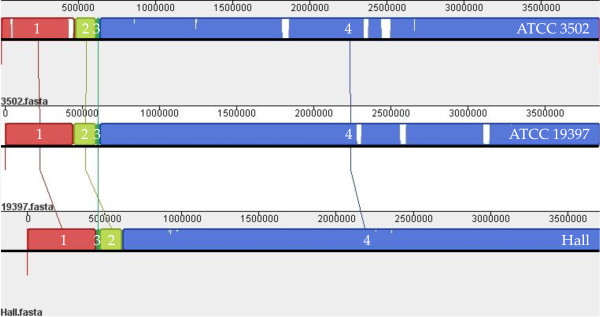
***C. botulinum *ATCC 3502, ATCC 19397, and Hall strains genome alignment**. The alignment display is organized into one horizontal "panel" per input genome sequence. Each genome's panel contains the name of the genome sequence, a scale showing the sequence coordinates for that genome, and a single black horizontal center line. All the blocks lie above the center line, which indicates that the aligned regions are in the forward orientation relative to the first genome sequence. Colored blocks in the first genome are connected by lines to similarly colored blocks in the second and third genomes. These lines indicate which regions in each genome are homologous and internally free from genomic rearrangement. There are only four blocks (1 to 4) from each genome. ATCC 3502 (top panel) and ATCC 19397 (middle panel) have the same block arrangements, while, in Hall (bottom panel), the positions of blocks 2 and 3 are switched. Inside each block, areas that are completely white were not aligned and contain sequence elements specific to a particular genome. Numbers in white inside the blocks indicate the block numbers.

As mentioned above, three fully sequenced *C. botulinum *A1 strain genomes are deposited in GenBank: ATCC 3502, ATCC 19397, and Hall. Mauve software [14] was used to compare and analyze the organization of these genomes. At the gross level, based on the ATCC 3502 genome organization, all three genomes were divided into four blocks: blocks 1, 2, 3, and 4, sequentially (Figure 3).

The three genomes were divided into two organizational patterns. ATCC 3502 and ATCC 19397 share the same pattern, while the positions of block 2 and block 3 were translocated in Hall, suggesting a genomic rearrangement event may have occurred among these strains. Moreover, within the same pattern and between genomes of ATCC 3502 and ATCC 19397, many regions inside the comparable blocks were different, as shown in areas that are completely white in Figure 3. Interestingly, two such regions (positions 1822680 through 1864850 and positions 2466354 through 2523055) in the ATCC 3502 genome are prophages that are absent in two other fully sequenced *C. botulinum *A1 strains: Hall and ATCC 19397 (data retrieved through NCBI Blast server). These observations are in agreement with previous reports [3,5] and also confirmed by our CGH findings (data not shown).

### Characterization of the ATCC 3502* and UMASS Hall strain genome organizational patterns

**Figure 4:**
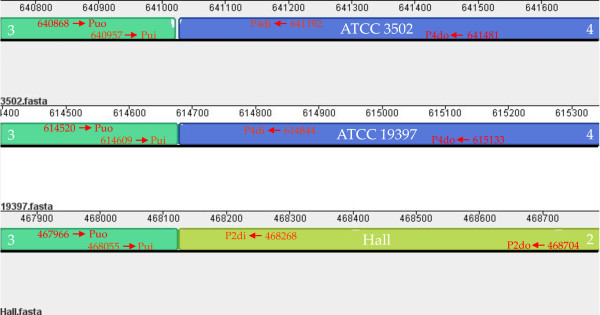
Primers designed to characterize the genome organizational patterns of ATCC 3502* and UMASS strains. This is part of exploded view of figure 3 on which the primer design is based. This region spans the boundary between block 3 and 4 (ATCC 3502 and ATCC 19397) and the boundary between block 3 and 2 (Hall). Puo, upstream outside primers; Pui, upstream inside primers; P4di, downstream inside primers for block 4; P4do, downstream outside primers for block 4; P2di, downstream inside primers for block 2, and P2do, downstream outside primers for block 2. Arrows with numbers in red indicate the starting (upstream primer) or ending (downstream primers) positions in each genome. Numbers in white inside the blocks indicate the block numbers.

**Figure 5:**
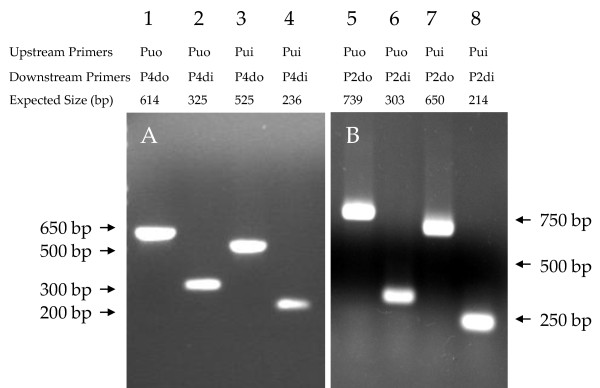
Identification of the genome organizational patterns of ATCC 3502* and UMASS strains by PCR. Panel A (lanes 1 through 4), PCR products from ATCC 3502* strain genomic DNA amplifications. Panel B (lanes 5 through 8), PCR products from UMASS strain genomic DNA amplifications.

To characterize whether the genome organizational pattern of ATCC 3502* and UMASS Hall strain fits into either of above two patterns, a PCR strategy was utilized. Primers were designed to span the boundary between block 3 and block 4 for the ATCC 3502 and ATCC 19397 pattern or between block 3 and block 2 for the Hall pattern (Figure 4). In one set of PCR reactions, using ATCC 3502* genomic DNA as template, the expected PCR product was generated from every PCR reaction with different combinations of upstream and downstream primers for ATCC 3502 and ATCC 19397 pattern (Figure 5 Panel A, lanes 1 to 4) but not from those with different combination of primers for Hall pattern reactions (data not shown). These results demonstrated that the genomic organization of ATCC 3502* indeed was identical to the ATCC 3502 Sanger Institute and ATCC 19397 strain genome organizations.

In the other set of PCR reactions, using UMASS strain genomic DNA as template, none of the PCR reactions containing one of four different primer combinations generated a product for ATCC 3502 and ATCC 19397 pattern (data not shown); however, every PCR reaction containing one of four different primer combinations amplified the predictable size of PCR products for Hall pattern (Figure 5 Panel B, lanes 5 to 8). The largest PCR product derived from PCR reaction with upstream primer (Puo) and downstream primer (P2do) combination and using UMASS strain genomic DNA as template was cloned and sequenced. The sequence from this product was 100% identical to the corresponding region in the Hall strain genome, confirming that the genomic organization of UMASS strain belonged to Hall type.

**Figure 6:**
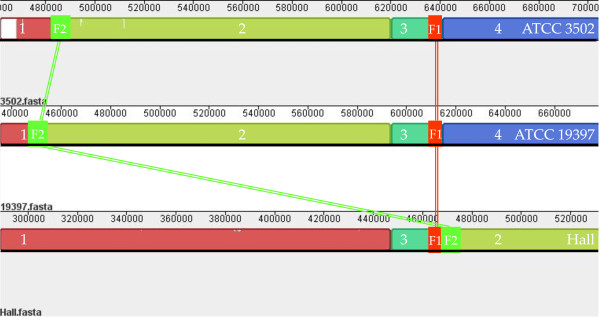
**PCR fragment distributions in *C. botulinum *type A1 strain genomes**. This is part of exploded view of figure 3. PCR product from Hall strain primers Puo and P2do combination was divided into two fragments: F1 (bright red) and F2 (bright green). F1 and F2 fragments were split in ATCC 3502 and ATCC 19397 genomes but remained within comparable blocks.

Analysis of the region containing the sequenced PCR product demonstrated that the region is further divided into F1 fragment (167 bp) that is located in 3'-end of block 3 of Hall strain genome and F2 fragment (587 bp) that is located in 5'-end of block 2 of Hall strain genome. As shown in Figure 6, the F1 and F2 fragments, a continuous region in Hall strain genome, were split into two separate fragments in ATCC 3502 and ATCC 19397 strain genomes, although each remained within their individual rearranged blocks.

**Figure 7:**
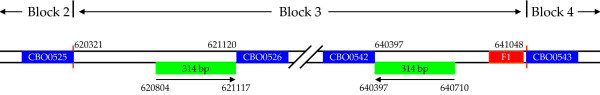
**Schematic representation of sequence elements of strain ATCC3502 genome block 3 and its flanking regions**. ATCC3502 genome block 3 spans the region between CBO0525 (the end of block 2) and CBO0543 (the start of block 4) and is 20728 bp in length. The vertical red lines indicate the block boundaries; the boxes in blue indicate annotated coding regions; the boxes in green indicate inverted repeat sequences; and the F1 fragment is indicated by a red box.

Further analysis of block 3 (20728 bp in strain ATCC 3502) revealed that virtually identical sequences are found in strain ATCC 19397 (20726 identities out of 20728 bp) and strain Hall (20710 identities out of 20714 bp). The GC content (27.3%) of block 3 in strain ATCC 3502 is not significantly different from 28.2% of whole genome GC content for each sequenced subtype A1 strain genome. Within block 3, we identified two 314 bp inverted repeat sequences (93% identities, Figure 7) that are located before the first gene in the block (CBO0526) and after the last gene in the block (CBO0542). Notably, no genes encoding a transposase or a direct repeat sequence (characteristic of transposon mobile element) was found in the region. In addition, the downstream inverted repeat has no overlapping sequence with F1 fragment mentioned above.

## Discussion

Genetic diversity has been described in other pathogenic bacterial species [15]. In one study, 73 *C. difficile *strains isolated from different resources were analyzed by CGH with microarrays containing coding sequences from *C. difficile *strains 630 and QCD-32g58. Startlingly, only about 16% of the genes in strain 630 were highly conserved among all strains [16]. In another study, comparison of the laboratory strain *Escherichia coli *K12 to both uropathogenic and enterohemorrhagic strains revealed that less than 40% of the total number of genes present were shared by these three strains [17]. Quite recently, CGH was performed on a relatively large scale to compare 61 strains of proteolytic *C. botulinum *and *C. sporogenes *using ATCC 3502 as reference strain [5]. Approximately 63% of the coding sequences (CDSs) present in reference strain ATCC 3502 were common to all 61 strains. Even within the toxin gene cluster, a typically conserved region, the gene arrangement could be different between different serotypes or subtypes of the same serotype [18,19]. The differences in the genome organization of the ATCC 3502* strain and ATCC 3502 (Sanger Institute), as shown in this report, further substantiated the dynamic nature of botulinum strain genome.

Lateral (or horizontal) gene transfer, through transformation, transduction, and conjugation, is a major mechanism for the generation of genetic diversity in pathogenic bacteria [20,21]. In *C. botulinum*, the neurotoxin cluster has been shown to be present within plasmids or on the chromosome in strains of the same or different serotypes, which is consistent with horizontal gene transfer [1]. None of the subtype A1 strains, whose genomes were sequenced, harbor the toxin gene on a plasmid. One plasmid, pBOT3502, existing in the ATCC 3502 Sanger Institute strain, was not found in the ATCC 19397 and Hall strains [3,5] and, even more strikingly, not in the ATCC 3502* strain genome sequences [18]. Further work is required to determine whether and if so, at what rate, loss of this plasmid occurs during laboratory passage.

The subtype A1 strain genetic diversity was also evidenced by the different location of genome block 3 when strains ATCC 3502 and ATCC 19397 are compared with strain Hall. Although this 20728 bp block contained two inverted repeat sequence fragments, we were unable to find direct repeat sequences or any gene that encodes a transposase. Therefore, we are unable to ascribe the genomic block switch observed in this study to a transposon-related mobile element mechanism [22-24]. Whether such differences in genomic arrangement among the subtype A1 strains examined has an effect on botulinum neurotoxin production remains to be elucidated.

In this report, the botulinum type A1 neurotoxin complex gene sequences of several strains were compared. There are at least five neurotoxin complex clusters from *C. botulinum *type A1 strains which have been fully sequenced and deposited into public databases. Sequence analysis showed that the sequences of five fully sequenced neurotoxin complex clusters were identical, and their gene coding regions and toxin gene complex from Allergan Hall strain displayed two synonymous single nucleotide polymorphisms: one is in the region encoding toxin heavy chain, the other in *botR*. These findings are quite different from those in an earlier report which showed that there were 93%, 94%, and 97% identities in the genes *ntnh*, *botR*, and *ha70 *at amino acid level, respectively [13]. The apparent discrepancy of these findings is likely due to different versions of genomic sequence that were used: version 16-Apr-2002 (GenBank accession number is unclear) of ATCC 3502 Hall strain was used in Allergan's report, while version 21-Nov-2006 (AM412317, which is one of the live versions) of ATCC 3502 Hall strain was used in this report.

## Conclusions

In summary, genetic diversity exists among the botulinum subtype A1 strains examined in this study. The neurotoxin gene of the UMASS strain exhibited the same nucleotide sequence as that of other published subtype A1 strains, except for the Allergan Hall strain. At the whole genome level, UMASS strain, ATCC3502*, and Sanger Institute 3502 strains, ATCC 19397, and Hall demonstrated differences in both gene content and genome arrangement.

## Methods

### Growth of bacterial strains

*C. botulinum *strains were grown anaerobically at 37°C in Trypticase-peptone-glucose-yeast extract (TPGY) medium. Stock cultures were stored in bovine brain medium at 4°C.

### Cloning and sequencing UMASS strain botulinum A1 toxin gene

PCR primers were designed to amplify the *C. botulinum *type A1 neurotoxin gene from UMASS strain. The PCR product was cloned and sequenced. The UMASS botulinum A1 neurotoxin nucleotide sequence, its counterpart regions in the Allergan Hall strain [13] and ATCC 3502 strain [GenBank: AM412317] were aligned by using EBI ClustalW2 http://www.ebi.ac.uk/Tools/clustalw2/index.html.

### Comparative genomic hybridization

Genomic DNA extraction was performed as described previously [18]. A custom *C. botulinum *type A1 strain ATCC 3502 comparative genomic hybridization arrays was used as described previously [18]. Genomic DNA from the UMASS test strain was labeled with Cy3 random primers and the reference strain, ATCC 3502*, was labeled with Cy5. The data were visualized with SignalMap version 1.9 (Nimblegen, Madison, WI) and are presented as normalized log_2 _ratios of the fluorescence intensity of the reference strain/test strain. The CGH microarray data were deposited in the NCBI Gene Expression Omnibus (GEO) [Accession: GSE21241]

### Whole genome comparison of *C. botulinum *A1 stains

Multiple genome alignments were performed by using Mauve [14]. Specifically, we analyzed the genome sequences of *C. botulinum *type A str. ATCC 3502 complete genome [GenBank: AM412317]; *C. botulinum *type A str. ATCC 19397 complete genome [GenBank: CP000726]; and *C. botulinum *type A str. Hall complete genome [GenBank: CP000727].

### Identification of ATCC 3502* and UMASS strain genome organizational patterns

Based on the genome organizational patterns observed by multiple genome alignment, PCR primers were designed in the way that the expected PCR fragment will span the boundaries between the rearranged block 3 (ATCC 3502 block number) and its surrounding blocks 2 and 4 for both patterns. The common upstream primers, which are inside the rearranged block 3, are 5'-GAA GGC CTC CGG TGG CGA TAT C-3' (outsider primers, Puo) and 5'-GTG TAG AGA ATC GAA ACA AAA TCA TCC ACA TC-3' (inside primer, Pui). The downstream primers inside the block 4 of ATCC 3502 and ATCC 19397 are 5'-CTT GAA TGG CTT GGC ATA TTA AGT GGG-3' (inside primer, P4di) and 5'-AGT TGG CTT TAT AAT CCC TTG GAT TTC AGG-3' (outsider primers, P4do). The downstream primers inside block 2 of Hall are 5'-CAG AAT TAG CAG ACA GAC TAC TTT CTA CC-3' (inside primer, P2di) and 5'-ATA GCC TTA TTT GGA GGC GGT CAG G-3' (outsider primers, P2do). Eight PCR reactions containing different upstream and downstream primer combinations were set up using genomic DNA isolated from either ATCC 3502* strain or UMASS strain. The PCR product amplified with primers Puo and P2do from the UMASS strain was cloned into pCR4-TOPO (Invitrogen, Carlsbad, CA) and sequenced, and the sequencing results were used to search the GenBank database.

## Authors' contributions

PKF drafted the manuscript and carried out part of experiments. BHR reviewed the manuscript and carried out part of experiments. BRS, SEM, and SC conceived, supervised, and coordinated the work; and reviewed the manuscript. All the authors read and approved the final manuscript.
